# Crystal structure of metobromuron

**DOI:** 10.1107/S205698901501347X

**Published:** 2015-07-22

**Authors:** Gihaeng Kang, Jineun Kim, Eunjin Kwon, Tae Ho Kim

**Affiliations:** a Department of Chemistry and Research Institute of Natural Sciences, Gyeongsang National University, Jinju 660-701, Republic of Korea

**Keywords:** crystal structure, metobromuron, phenyl­urea herbicide, hydrogen bonding, Br⋯Br contacts

## Abstract

The title compound [systematic name: 3-(4-bromo­phen­yl)-1-meth­oxy-1-methyl­urea], C_9_H_11_BrN_2_O_2_, is a phenyl­urea herbicide. The dihedral angle between the plane of the urea group and that of the bromo­phenyl ring is 39.13 (10)°. In the crystal, N—H⋯O and C—H⋯O hydrogen bonds and weak C—H⋯π inter­actions link adjacent mol­ecules, forming chains along the *a*-axis direction. In addition, short inter­molecular Br⋯Br contacts [3.648 (4) Å] are present, resulting in a two-dimensional network extending parallel to (101).

## Related literature   

For information on the herbicidal properties of the title compound, see: Leila *et al.* (2011 ▸). For related crystal structures, see: Black *et al.* (2010 ▸); Kostyanovsky *et al.* (2010 ▸).
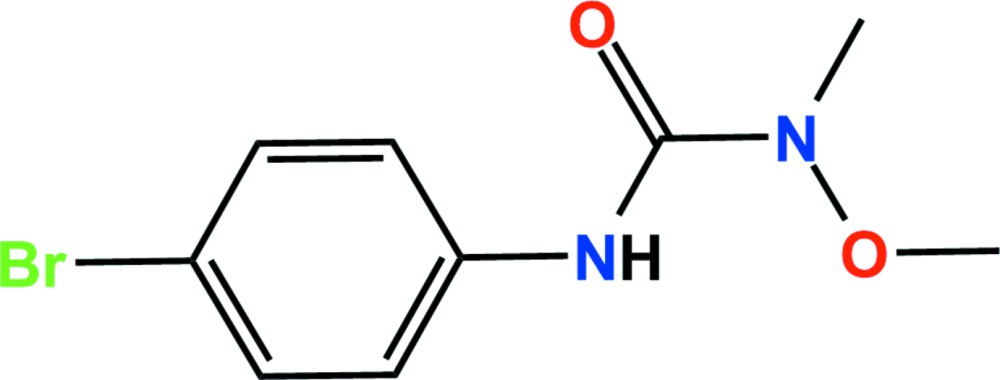


## Experimental   

### Crystal data  

C_9_H_11_BrN_2_O_2_
*M* *_r_* = 259.11
Orthorhombic, 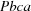

*a* = 9.8184 (2) Å
*b* = 11.3286 (3) Å
*c* = 18.9569 (5) Å
*V* = 2108.55 (9) Å^3^
*Z* = 8
Mo *K*α radiation
μ = 3.88 mm^−1^
*T* = 173 K
0.30 × 0.16 × 0.02 mm

### Data collection  

Bruker APEXII CCD diffractometer
Absorption correction: multi-scan (*SADABS*; Bruker, 2013 ▸) *T*_min_ = 0.389, *T*_max_ = 0.927
17922 measured reflections
2424 independent reflections
1857 reflections with *I* > 2σ(*I*)
*R*_int_ = 0.048

### Refinement  

*R*[*F* ^2^ > 2σ(*F* ^2^)] = 0.029
*wR*(*F* ^2^) = 0.066
*S* = 1.03
2424 reflections
129 parameters
H-atom parameters constrained
Δρ_max_ = 0.41 e Å^−3^
Δρ_min_ = −0.42 e Å^−3^

### 

Data collection: *APEX2* (Bruker, 2013 ▸); cell refinement: *SAINT* (Bruker, 2013 ▸); data reduction: *SAINT*; program(s) used to solve structure: *SHELXS97* (Sheldrick 2008 ▸); program(s) used to refine structure: *SHELXL2013* (Sheldrick, 2015 ▸); molecular graphics: *DIAMOND* (Brandenburg, 2010 ▸); software used to prepare material for publication: *SHELXTL* (Sheldrick, 2008 ▸).

## Supplementary Material

Crystal structure: contains datablock(s) global, I. DOI: 10.1107/S205698901501347X/hg5451sup1.cif

Structure factors: contains datablock(s) I. DOI: 10.1107/S205698901501347X/hg5451Isup2.hkl

Supporting information file. DOI: 10.1107/S205698901501347X/hg5451Isup3.cml

**Fig. 1.**
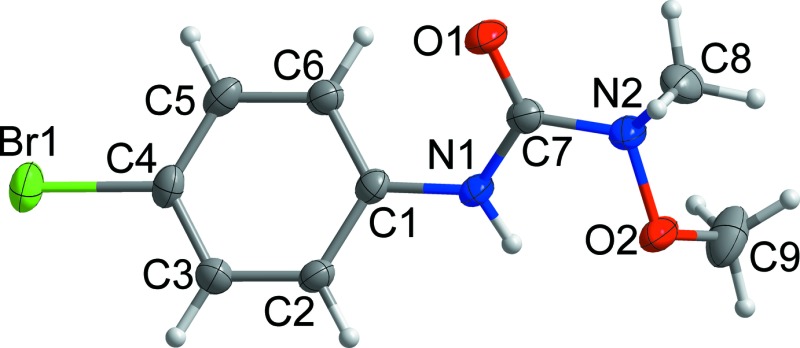
The asymmetric unit of the title compound with the atom numbering scheme. Displacement ellipsoids are drawn at the 50% probability level. H atoms are shown as small spheres of arbitrary radius.

**Fig. 2.**
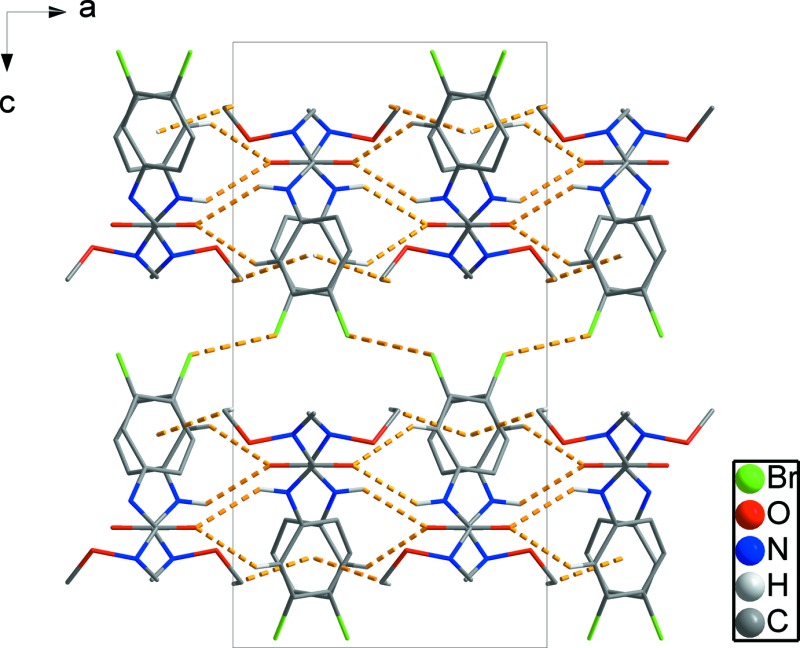
Crystal packing viewed along the b axis. The intermolecular interactions are shown as dashed lines.

. DOI: 10.1107/S205698901501347X/hg5451fig1.tif
The asymmetric unit of the title compound with the atom numbering scheme. Displacement ellipsoids are drawn at the 50% probability level. H atoms are shown as small spheres of arbitrary radius.

b . DOI: 10.1107/S205698901501347X/hg5451fig2.tif
Crystal packing viewed along the *b* axis. The inter­molecular inter­actions are shown as dashed lines.

CCDC reference: 1412609

Additional supporting information:  crystallographic information; 3D view; checkCIF report

## Figures and Tables

**Table 1:** Hydrogen-bond geometry (, ) *Cg*1 is the centroid of the C1C6 ring.

*D*H*A*	*D*H	H*A*	*D* *A*	*D*H*A*
N1H1*N*O1^i^	0.88	2.39	3.130(2)	142
C2H2O1^i^	0.95	2.42	3.217(3)	142
C9H9*A* *Cg*1^i^	0.98	2.99	3.477(3)	112

Symmetry code: (i) 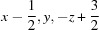.

## supplementary crystallographic information

### S1. Comment

Metobromuron: 3-(4-bromophenyl)-1-methoxy-1-methylurea, is a phenylurea herbicide and has been used for the control of broadleaf weeds in cereal and vegetable crops, acting through the inhibition of photosynthesis (Leila *et al.*, 2011). However, until now its crystal structure has not been reported. In the title compound (Fig. 1), the dihedral angle between the plane of the urea group and the bromophenyl ring is 39.13 (10) °. All bond lengths and bond angles are normal and comparable to those observed in similar crystal structures (Black *et al.*, 2010; Kostyanovsky *et al.*, 2010).

In the crystal structure (Fig. 2), N–H···O and C–H···O hydrogen bonds and weak C–H···π interactions link adjacent molecules (Table 1), forming one-dimensional chains along the *a*-axis direction. In addition, weak intermolecular short Br···Br contacts [3.648 (4) Å] are present, resulting in a two-dimensional network extending parallel to (101).

### S2. Experimental

The title compound was purchased from the Dr. Ehrenstorfer GmbH Company. Slow evaporation of a solution in ethyl acetate gave single crystals suitable for X-ray analysis.

### S3. Refinement

All H-atoms were positioned geometrically and refined using a riding model with d(N—H) = 0.88 Å, *U*_iso_ = 1.2*U*_eq_(C) for urea N—H, d(C—H) = 0.98 Å, *U*_iso_ = 1.5*U*_eq_(C) for methyl group, d(C—H) = 0.95 Å, *U*_iso_ = 1.2*U*_eq_(C) for aromatic C—H.

### Crystal data

**Table:**

C_9_H_11_BrN_2_O_2_	*D*_x_ = 1.632 Mg m^−^^3^
*M**_r_* = 259.11	Mo *K*α radiation, λ = 0.71073 Å
Orthorhombic, *P**b**c**a*	Cell parameters from 3544 reflections
*a* = 9.8184 (2) Å	θ = 3.0–24.1°
*b* = 11.3286 (3) Å	µ = 3.88 mm^−^^1^
*c* = 18.9569 (5) Å	*T* = 173 K
*V* = 2108.55 (9) Å^3^	Plate, colourless
*Z* = 8	0.30 × 0.16 × 0.02 mm
*F*(000) = 1040	

### Data collection

**Table:**

Bruker APEXII CCD diffractometer	1857 reflections with *I* > 2σ(*I*)
φ and ω scans	*R*_int_ = 0.048
Absorption correction: multi-scan (*SADABS*; Bruker, 2013)	θ_max_ = 27.5°, θ_min_ = 2.2°
*T*_min_ = 0.389, *T*_max_ = 0.927	*h* = −12→12
17922 measured reflections	*k* = −14→14
2424 independent reflections	*l* = −23→24

### Refinement

**Table:**

Refinement on *F*^2^	0 restraints
Least-squares matrix: full	Hydrogen site location: inferred from neighbouring sites
*R*[*F*^2^ > 2σ(*F*^2^)] = 0.029	H-atom parameters constrained
*wR*(*F*^2^) = 0.066	*w* = 1/[σ^2^(*F*_o_^2^) + (0.0255*P*)^2^ + 1.105*P*] where *P* = (*F*_o_^2^ + 2*F*_c_^2^)/3
*S* = 1.03	(Δ/σ)_max_ = 0.001
2424 reflections	Δρ_max_ = 0.41 e Å^−^^3^
129 parameters	Δρ_min_ = −0.42 e Å^−^^3^

### Special details

**Table:**

Geometry. All e.s.d.'s (except the e.s.d. in the dihedral angle between two l.s. planes) are estimated using the full covariance matrix. The cell e.s.d.'s are taken into account individually in the estimation of e.s.d.'s in distances, angles and torsion angles; correlations between e.s.d.'s in cell parameters are only used when they are defined by crystal symmetry. An approximate (isotropic) treatment of cell e.s.d.'s is used for estimating e.s.d.'s involving l.s. planes.

### Fractional atomic coordinates and isotropic or equivalent isotropic displacement parameters (Å^2^)

**Table:**

	*x*	*y*	*z*	*U*_iso_*/*U*_eq_	
Br1	0.86464 (3)	0.10694 (2)	0.51608 (2)	0.03673 (10)	
O1	0.88255 (14)	0.47320 (14)	0.80131 (9)	0.0337 (4)	
O2	0.55297 (15)	0.56454 (15)	0.83862 (9)	0.0338 (4)	
N1	0.67399 (18)	0.41832 (16)	0.75709 (10)	0.0259 (4)	
H1N	0.5855	0.4269	0.7624	0.031*	
N2	0.69171 (18)	0.54270 (17)	0.85274 (10)	0.0297 (5)	
C1	0.7206 (2)	0.34339 (18)	0.70276 (12)	0.0224 (5)	
C2	0.6508 (2)	0.34425 (19)	0.63907 (12)	0.0249 (5)	
H2	0.5732	0.3934	0.6335	0.030*	
C3	0.6936 (2)	0.2741 (2)	0.58381 (12)	0.0276 (5)	
H3	0.6467	0.2756	0.5400	0.033*	
C4	0.8053 (2)	0.20197 (19)	0.59297 (12)	0.0265 (5)	
C5	0.8734 (2)	0.19632 (19)	0.65655 (12)	0.0278 (5)	
H5	0.9485	0.1444	0.6625	0.033*	
C6	0.8306 (2)	0.26736 (19)	0.71147 (12)	0.0264 (5)	
H6	0.8766	0.2643	0.7555	0.032*	
C7	0.7577 (2)	0.47817 (19)	0.80181 (12)	0.0248 (5)	
C8	0.7580 (3)	0.6412 (2)	0.88719 (14)	0.0378 (6)	
H8A	0.8452	0.6152	0.9069	0.057*	
H8B	0.6996	0.6709	0.9252	0.057*	
H8C	0.7740	0.7042	0.8528	0.057*	
C9	0.4734 (3)	0.5087 (3)	0.89159 (16)	0.0525 (8)	
H9A	0.4974	0.4249	0.8941	0.079*	
H9B	0.3766	0.5168	0.8800	0.079*	
H9C	0.4914	0.5461	0.9373	0.079*	

### Atomic displacement parameters (Å^2^)

**Table:**

	*U*^11^	*U*^22^	*U*^33^	*U*^12^	*U*^13^	*U*^23^
Br1	0.04164 (16)	0.03414 (14)	0.03440 (17)	0.00799 (11)	0.01142 (11)	−0.00470 (11)
O1	0.0172 (9)	0.0434 (10)	0.0404 (10)	0.0032 (7)	−0.0014 (7)	−0.0051 (8)
O2	0.0191 (8)	0.0430 (10)	0.0395 (10)	0.0057 (7)	−0.0011 (7)	−0.0108 (8)
N1	0.0157 (9)	0.0335 (11)	0.0285 (11)	0.0028 (7)	0.0012 (8)	−0.0047 (9)
N2	0.0195 (10)	0.0382 (12)	0.0314 (12)	0.0014 (8)	−0.0023 (8)	−0.0082 (9)
C1	0.0194 (11)	0.0238 (11)	0.0241 (12)	0.0008 (8)	0.0040 (9)	0.0012 (9)
C2	0.0203 (12)	0.0278 (11)	0.0267 (13)	0.0060 (9)	0.0009 (9)	0.0021 (10)
C3	0.0275 (12)	0.0311 (12)	0.0241 (13)	0.0037 (9)	−0.0005 (10)	0.0014 (10)
C4	0.0282 (12)	0.0256 (11)	0.0255 (13)	0.0023 (9)	0.0092 (10)	0.0001 (10)
C5	0.0229 (12)	0.0254 (11)	0.0350 (14)	0.0057 (9)	0.0063 (10)	0.0046 (10)
C6	0.0235 (12)	0.0301 (12)	0.0254 (12)	0.0034 (9)	0.0009 (9)	0.0033 (10)
C7	0.0223 (12)	0.0273 (11)	0.0249 (12)	0.0011 (9)	0.0003 (9)	0.0032 (9)
C8	0.0338 (14)	0.0426 (15)	0.0371 (15)	−0.0010 (11)	−0.0087 (12)	−0.0110 (12)
C9	0.0383 (16)	0.0555 (18)	0.064 (2)	−0.0075 (13)	0.0224 (15)	−0.0177 (16)

### Geometric parameters (Å, º)

**Table:**

Br1—C4	1.904 (2)	C3—C4	1.378 (3)
O1—C7	1.228 (3)	C3—H3	0.9500
O2—N2	1.410 (2)	C4—C5	1.380 (3)
O2—C9	1.421 (3)	C5—C6	1.381 (3)
N1—C7	1.361 (3)	C5—H5	0.9500
N1—C1	1.411 (3)	C6—H6	0.9500
N1—H1N	0.8800	C8—H8A	0.9800
N2—C7	1.373 (3)	C8—H8B	0.9800
N2—C8	1.448 (3)	C8—H8C	0.9800
C1—C2	1.388 (3)	C9—H9A	0.9800
C1—C6	1.391 (3)	C9—H9B	0.9800
C2—C3	1.380 (3)	C9—H9C	0.9800
C2—H2	0.9500		
			
N2—O2—C9	108.60 (19)	C4—C5—H5	120.5
C7—N1—C1	123.95 (18)	C6—C5—H5	120.5
C7—N1—H1N	118.0	C5—C6—C1	120.5 (2)
C1—N1—H1N	118.0	C5—C6—H6	119.8
C7—N2—O2	114.55 (17)	C1—C6—H6	119.8
C7—N2—C8	121.04 (19)	O1—C7—N1	125.1 (2)
O2—N2—C8	112.62 (18)	O1—C7—N2	120.1 (2)
C2—C1—C6	119.4 (2)	N1—C7—N2	114.73 (19)
C2—C1—N1	118.07 (19)	N2—C8—H8A	109.5
C6—C1—N1	122.5 (2)	N2—C8—H8B	109.5
C3—C2—C1	120.4 (2)	H8A—C8—H8B	109.5
C3—C2—H2	119.8	N2—C8—H8C	109.5
C1—C2—H2	119.8	H8A—C8—H8C	109.5
C4—C3—C2	119.2 (2)	H8B—C8—H8C	109.5
C4—C3—H3	120.4	O2—C9—H9A	109.5
C2—C3—H3	120.4	O2—C9—H9B	109.5
C3—C4—C5	121.5 (2)	H9A—C9—H9B	109.5
C3—C4—Br1	118.85 (18)	O2—C9—H9C	109.5
C5—C4—Br1	119.62 (16)	H9A—C9—H9C	109.5
C4—C5—C6	119.0 (2)	H9B—C9—H9C	109.5
			
C9—O2—N2—C7	116.7 (2)	Br1—C4—C5—C6	178.85 (16)
C9—O2—N2—C8	−99.8 (2)	C4—C5—C6—C1	0.0 (3)
C7—N1—C1—C2	−141.5 (2)	C2—C1—C6—C5	2.3 (3)
C7—N1—C1—C6	40.2 (3)	N1—C1—C6—C5	−179.4 (2)
C6—C1—C2—C3	−2.8 (3)	C1—N1—C7—O1	−1.2 (4)
N1—C1—C2—C3	178.9 (2)	C1—N1—C7—N2	−177.6 (2)
C1—C2—C3—C4	0.9 (3)	O2—N2—C7—O1	165.9 (2)
C2—C3—C4—C5	1.4 (3)	C8—N2—C7—O1	25.7 (3)
C2—C3—C4—Br1	−179.30 (17)	O2—N2—C7—N1	−17.6 (3)
C3—C4—C5—C6	−1.9 (3)	C8—N2—C7—N1	−157.8 (2)

### Hydrogen-bond geometry (Å, º)

Cg1 is the centroid of the C1–C6 ring.

**Table:**

*D*—H···*A*	*D*—H	H···*A*	*D*···*A*	*D*—H···*A*
N1—H1*N*···O1^i^	0.88	2.39	3.130 (2)	142
C2—H2···O1^i^	0.95	2.42	3.217 (3)	142
C9—H9*A*···*Cg*1^i^	0.98	2.99	3.477 (3)	112

Symmetry code: (i) *x*−1/2, *y*, −*z*+3/2.
