# Supplementary figures and images for: Crystal structure of metobromuron
Source: Acta Crystallogr E Crystallogr Commun. 2015 Jul 22;71(Pt 8):o589. doi: 10.1107/S205698901501347X (PMC4571412; doi:10.1107/S205698901501347X)

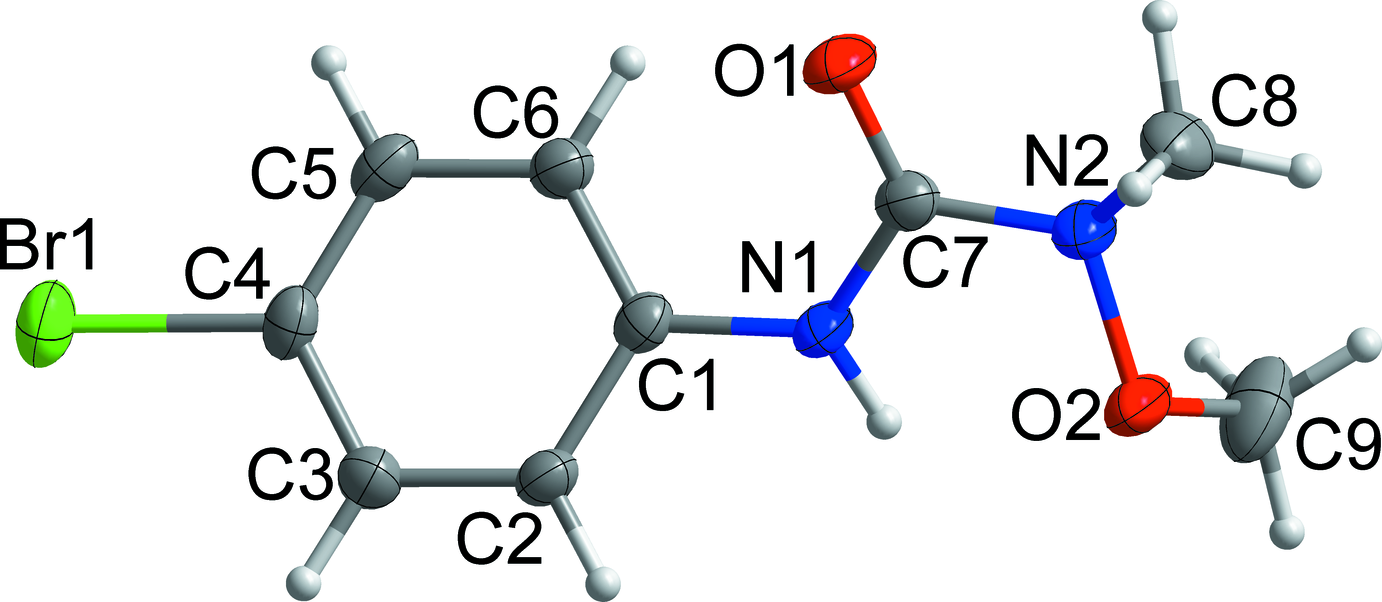

Supplement: Supplementary file 4 [file e-71-0o589-fig1.tif]

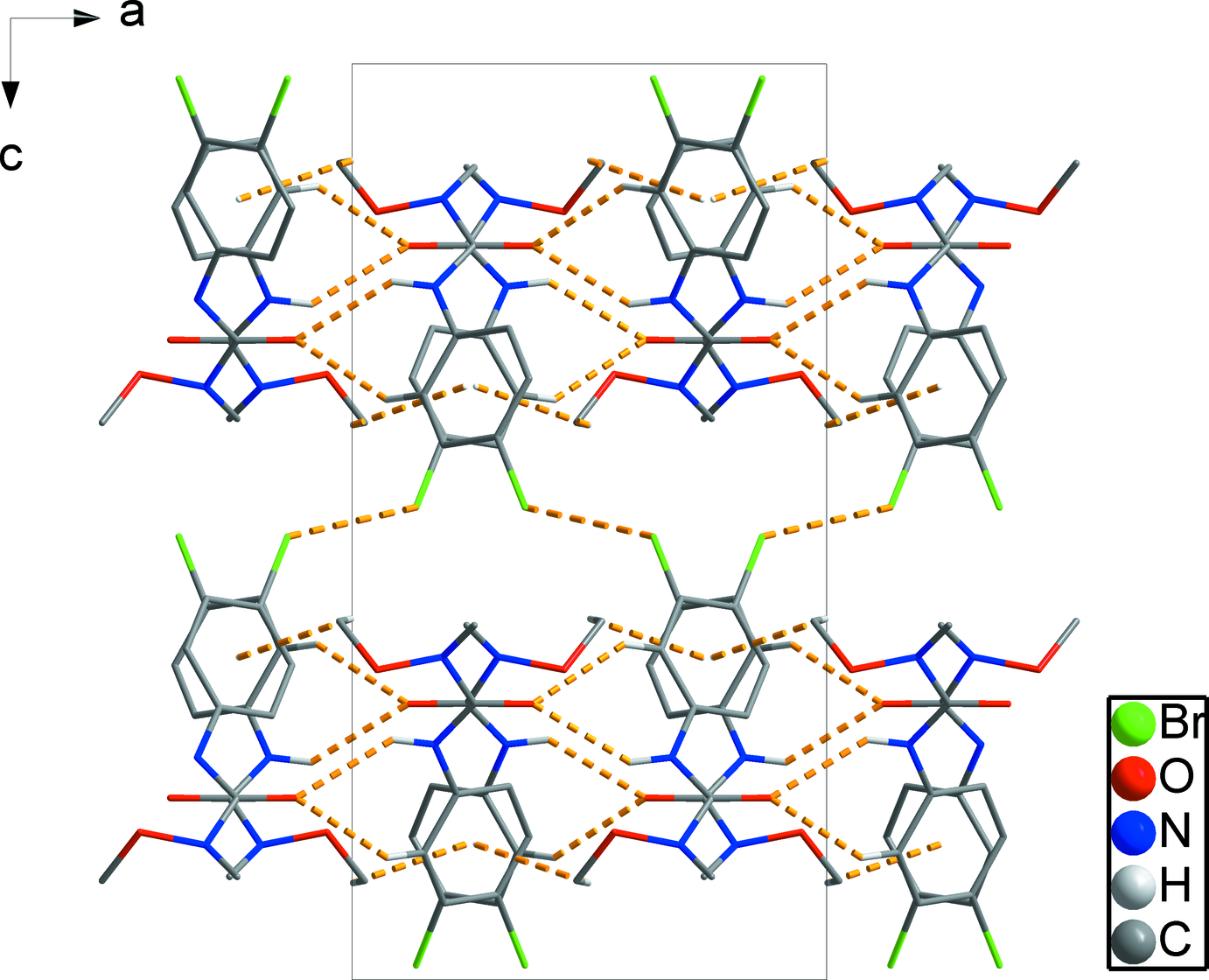

Supplement: Supplementary file 5 [file e-71-0o589-fig2.tif]
